# Serum Vitamin Levels and Their Relationships with Other Biomarkers in Korean Breast Cancer Patients

**DOI:** 10.3390/nu12092831

**Published:** 2020-09-16

**Authors:** Jee Ah Kim, Rihwa Choi, Hojeong Won, Seonwoo Kim, Hee Jun Choi, Jai Min Ryu, Se Kyung Lee, Jonghan Yu, Seok Won Kim, Jeong Eon Lee, Soo-Youn Lee

**Affiliations:** 1 Department of Laboratory Medicine and Genetics, Samsung Medical Center, Sungkyunkwan University School of Medicine, 81 Irwon-ro, Gangnam-gu, Seoul 06351, Korea; jeeah89.kim@samsung.com (J.A.K.); pirate0720@naver.com (R.C.); 2 Department of Laboratory Medicine, Green Cross Laboratories, Gyeonggi, Yongin 16924, Korea; 3 Statistics and Data Center, Research Institute for Future Medicine, Samsung Medical Center, Seoul 06351, Korea; hojeong.won@sbri.co.kr (H.W.); seonwoo.kim@samsung.com (S.K.); 4 Department of Surgery, Samsung Changwon Hospital, Sungkyunkwan University School of Medicine, Changwon 51353, Korea; heejun1.choi@samsung.com; 5 Division of Breast Surgery, Department of Surgery, Samsung Medical Center, Sungkyunkwan University School of Medicine, 81 Irwon-ro, Gangnam-gu, Seoul 06351, Korea; jaimin.ryu@samsung.com (J.M.R.); sekyung.lee@samsung.com (S.K.L.); lymbics@hanmail.net (J.Y.); seokwon1.kim@samsung.com (S.W.K.)

**Keywords:** vitamin, benign breast disease, breast cancer, Korea

## Abstract

Numerous studies have shown that vitamins reduce the risk of cancers, but the relationship between serum vitamin levels and breast cancer is still controversial. In this study, we evaluated serum levels of vitamins in Korean patients with benign breast disease or breast cancer and investigated their associations with clinical and laboratory parameters. Concentrations of vitamin A, D, and E, together with homocysteine and methylmalonic acid as biomarkers of vitamin B12 deficiency, were measured by high-performance liquid chromatography (HPLC) or liquid chromatography with tandem mass spectrometry (LC-MS/MS) in the serum of 104 breast cancer patients, 62 benign breast disease patients, and 75 healthy Korean females. We further assessed possible associations between vitamin levels and breast cancer subtypes, the presence of lymph node metastasis, and tumor stages. Serum concentrations of vitamins A and E were significantly lower in breast cancer patients and in benign breast disease patients than in healthy controls. Severe vitamin D deficiency was more prevalent in breast cancer patients than in healthy controls. Vitamin D level was significantly lower in breast cancer patients with estrogen receptor-negative or triple-negative subtypes than in those with other subtypes. Further research with a larger study population is required to elucidate the role of vitamins in breast cancer.

## 1. Introduction

Environmental factors such as radiation, pollution, infection, and diet contribute significantly to the onset of cancer. Numerous studies have identified the positive effects of dietary patterns on reducing certain types of cancer [1]. Through epidemiological studies, a role for nutrients in cancer development has been suggested and is associated to the antioxidant properties. Certain nutrients affect DNA repair, inflammation, and levels of endogenous hormones and growth factors by regulating gene expression [2].

Vitamins are required by various physiological and biochemical mechanisms of the body and are known to have antioxidant properties and improve the immune response [3]. A number of studies have shown that low intake and low serum vitamin levels have a strong association with a higher risk of cancer [4,5,6,7]. Over the past two decades, researchers have focused on the roles of vitamin in breast cancer etiology [8,9,10]. Some studies have reported a relationship between dietary pattern and reduced risk of postmenopausal breast cancer [11,12]. However, results among studies are often conflicting and much remains unknown about the relationship between vitamins and breast cancer. In addition, because benign breast disease is associated with increased breast cancer risk [13,14], there are efforts to identify the relationship between serum vitamin levels and benign breast disease, but results are inconsistent and inconclusive [15,16]. Possible reasons for inconsistencies among studies may be the use of different analytical methods to measure various metabolites of the vitamins of interest, and enrollment of participants from populations of different ethnicities.

Dietary factors may have different impacts according to breast cancer subtype, but only vitamin D has been investigated in this context. As specific nutrients and vitamins may be more particularly related to the promotion of, or protection from, certain types of breast cancer, stratification of breast cancer by specific tumor characteristics such as tumor stage and molecular classification needs to be considered [17,18].

In this study, we evaluated serum levels of multiple vitamins in Korean female patients with benign breast disease and patients with breast cancer and investigated their relationships with clinical/laboratory parameters. We further evaluated possible associations between serum concentrations of vitamin markers and breast cancer subtypes, the presence of lymph node metastasis, and tumor stages, as these phenotypes have significant implications for prognosis and the effectiveness of targeted therapies.

## 2. Materials and Methods

### 2.1. Study Population

We performed a case-control study comprising 104 adult female patients histopathologically diagnosed with breast cancer, 62 female patients with a histopathological diagnosis of benign breast disease, including fibrocystic change or fibroadenoma, and 75 healthy Korean females. All patients with breast cancer or breast disease were diagnosed at Samsung Medical Center (Seoul, Korea), a tertiary care hospital, between March 2015 and March 2019. Patients with any tumor other than primary breast benign or malignant tumor, patients with malabsorption, patients with recurrent infections, patients with inflammatory diseases, or patients with impaired liver or kidney function (total bilirubin >2.5 mg/dL, aspartate aminotransferase (AST) or alanine aminotransferase (ALT) >3 times the upper limit of the reference interval, alkaline phosphatase (ALP) >5 times the upper limit of the reference interval, serum creatinine >1.8 mg/dL) were excluded. The 75 control female subjects with no benign breast disease or breast cancer were recruited from among individuals who visited a health promotion center for a medical checkup who had no clinical symptoms or signs of breast disease.

The study was approved by the Institutional Review Board of Samsung Medical Center (IRB No: SMC-2016-07-129). Demographic and clinical characteristics including age, body mass index (BMI), menopausal status and serum biochemistry test results (total protein, albumin, AST, ALT, ALP, high density lipoprotein (HDL), low density lipoprotein (LDL), total cholesterol) were collected from electronic medical records. Individuals were classified as overweight if 23 ≤ BMI < 25 kg/m^2^ and as obese if BMI ≥ 25 kg/m^2^ in accordance with World Health Organization (WHO) guidelines for Asian populations [19]. Information obtained from surgery about breast cancer subtypes such as estrogen receptor (ER), progesterone receptor (PR), and human epidermal growth factor receptor 2 (HER2) presence or absence was reviewed retrospectively through electronic medical records, in addition to tumor stage and the presence of lymph node metastasis.

### 2.2. Analytical Procedures

Blood samples to determine vitamin levels were collected from patients who were fasted during the first visit before any treatment. Serum levels of vitamin A and E were measured by high-performance liquid chromatography (HPLC). Serum vitamin D (25(OH)D) level was determined as the sum of serum 25-hydroxyvitamin D2 (25(OH)D_2_) and 25-hydroxyvitamin D3 (25(OH)D_3_) levels. Liquid chromatography with tandem mass spectrometry (LC-MS/MS) was used to measure serum 25(OH)D_2_ and 25(OH)D_3_ levels (Figure S1). Serum homocysteine and methylmalonic acid levels, as indicators of vitamin B12 status, were measured by LC-MS/MS. All serum levels of vitamin markers were measured using methods reported by Oh et al. [20]. The accuracy of serum vitamin A, D, E, and B12 indicator measurements were verified by regularly participating in external quality assurance programs including the Proficiency Testing/Quality Management program of the College of American Pathologists (CAP) survey and the Vitamin D External Quality Assessment Scheme (DEQAS). Coefficients of variation for intra- and inter-assay were <10% in all assays, indicating good repeatability. To evaluate biochemical status, serum chemistry parameters such as total protein, albumin, AST, ALT, ALP, HDL, LDL, and total cholesterol were measured using a Roche cobas c702 clinical analyzer (Roche Diagnostics Corp., Indianapolis, IN, USA).

Vitamin deficiencies were defined as described in previous reports: vitamin A deficiency as <1.05 µmol/L, vitamin E deficiency as <11.6 µmol/L, and vitamin B12 deficiency as a methylmalonic acid concentration >300 nmol/L or a homocysteine concentration >15 µmol/L [20]. Vitamin D deficiency was defined as a serum 25(OH)D concentration <20 ng/mL and severe vitamin D deficiency was defined as a serum 25(OH)D concentration <10 ng/mL [21].

### 2.3. Statistical Analysis

SPSS software v25.0 (SPSS Inc. 233 S. Chicago, IL, USA) was used to analyze data. Continuous variables are presented as medians and interquartile ranges (IQRs). *p*-values less than 0.05 were considered to be statistically significant. We used the Kruskal–Wallis test, ANOVA, and Tukey’s test on ranked data to assess the significance of differences in serum levels of vitamin status markers and vitamin deficiencies among breast cancer patients, benign breast disease patients, and healthy controls. Odds ratios and 95% confidence intervals (CI) were calculated to estimate relative risks for the exposure variables directly of interest as well as potential confounding variables. Potential confounding effects of age, menopausal state, BMI, total protein, albumin, AST, ALT, ALP, and total cholesterol were adjusted for in multivariate logistic regression analysis. Wilcoxon rank sum test and Kruskal–Wallis test were used to analyze the significance of differences in breast cancer subtypes. To evaluate the associations among serum levels of vitamin status markers, demographic data, and biochemical results, Spearman’s correlations were calculated. According to the classification system which Hebel et al. proposed, *r* values below 0.2 indicate a negligible correlation, *r* values between 0.2 and 0.5 indicate a weak positive correlation, *r* values between 0.5 and 0.8 indicate a moderate positive correlation, and *r* values between 0.8 and 1.0 indicate a strong positive correlation [22].

## 3. Results

### 3.1. General Characteristics of the Study Population

This study comprised 75 healthy controls, 62 patients with benign breast disease, and 104 patients with breast cancer. The general characteristics of the study participants are summarized in Table 1. Patients with breast cancer had a higher BMI than the other groups (*p* = 0.0059) and had a high prevalence of obesity compared to healthy controls and patients with benign breast disease (*p* = 0.0053). Breast cancer patients, as well as benign breast disease patients, had significantly lower concentrations of total cholesterol than healthy controls (*p* = 0.0004). Among the 104 breast cancer patients, 92 (88.5%) had ER-positive breast cancer, 85 (81.7%) had PR-positive breast cancer, 28 (26.9%) had HER2-positive/equivocal breast cancer, while six (5.8%) had triple negative breast cancer. Sixteen (15.4%) patients with breast cancer exhibited lymph node metastasis.

### 3.2. Vitamin Status in the Study Population

**Figure 1:**
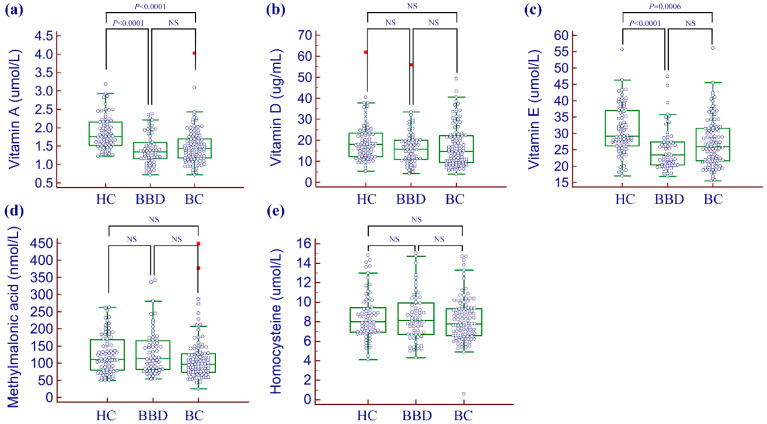
Comparison of vitamins and vitamin B12 status indicators between healthy controls, patients with benign breast disease, and patients with breast cancer. (**a**) vitamin A, (**b**) vitamin D, (**c**) vitamin E, (**d**) methylmalonic acid, and (**e**) homocysteine. Abbreviations: NS, not significant; HC, healthy controls; BBD, patients with benign breast disease; BC, patients with breast cancer.

Serum levels of vitamins and vitamin biomarkers in the study populations are presented in Table 2 and Figure 1. Serum concentrations of vitamins A and E in patients with breast cancer and in patients with benign breast disease were significantly lower than in healthy controls, respectively (*p* < 0.001). However, serum concentrations of vitamin D, homocysteine, and methylmalonic acid were not statistically different between groups. There were no statistically significant differences among serum vitamin levels between patients with breast cancer and patients with benign breast disease. The odds of having benign or malignant breast tumors were estimated through multivariable-adjusted logistic regression models (Table 3). Vitamin A was associated with a lower risk of both benign breast disease (odds ratio (OR) = 0.13, 95% CI = 0.04–0.36, *p* = 0.0001) and breast cancer (OR = 0.20, 95% CI = 0.08–0.48, *p* = 0.0004). Vitamins D, E, methylmalonic acid, and homocysteine showed no significant associations with either benign breast disease or breast cancer.

Vitamin A deficiency (<1.05 μmol/L) was observed in 13 patients with breast cancer (12.5%), and in 11 patients with benign breast disease (17.7%), but in none of the healthy controls. Severe vitamin D deficiency (<10 ng/mL) was significantly more prevalent in patients with breast cancer than in patients with benign breast disease and in healthy controls (29/104 (27.9%) in breast cancer patients vs. 12/62 (19.4%) in benign breast disease patients vs. 7/75 (9.3%) in healthy controls, *p* = 0.0092). Vitamin B12 deficiency (methylmalonic acid >300 nmol/L or homocysteine >15 μmol/L) was observed in two patients with breast cancer (1.9%), and in two patients with benign breast disease (3.2%), but not in any healthy controls. No patients had vitamin E deficiency (<11.6 μmol/L).

Serum levels of vitamins were compared among breast cancer patients with or without ER, PR, HER2, and triple-negative breast cancer which were negative for all of three receptors (Table 4). Concentrations of vitamin D were significantly lower in ER-negative breast cancer patients than in ER-positive breast cancer patients (*p* = 0.0233). In women with triple-negative breast cancer, concentrations of vitamin D were significantly lower (*p* = 0.0266) and concentrations of methylmalonic acid was significantly higher (*p* = 0.0384) than in women with breast cancer that expresses one or more of the hormone receptors. Serum levels of vitamins were compared among breast cancer patients with and without nodal metastasis and different stages of breast cancer, but no significant differences were observed.

### 3.3. Correlations among Vitamin Markers and Biochemical Factors

The correlations among serum concentrations of vitamins and biochemical parameters were analyzed (Table 5). Vitamin E concentration showed a weak positive correlation with LDL (*r* = 0.42, *p* < 0.0001) and a moderate positive correlation with total cholesterol (*r* = 0.586, *p* < 0.0001).

## 4. Discussion

In this study, we measured serum levels of vitamins in female Korean patients with breast cancer or benign breast disease, and in healthy controls. We also analyzed possible relationships between serum levels of vitamins and molecular subtypes of breast cancer, the presence of lymph node metastasis, and tumor stages. The present study is one of only a few studies to investigate serum levels of multiple vitamins and to examine the association between serum levels of vitamins and breast cancer in an Asian population.

As previously reported, our study results showed that patients with breast cancer were considered to be more frequently obese than healthy controls or patients with benign breast disease. Obesity increases the risk of postmenopausal breast cancer, since adipose tissue enhances the mammary estrogen signaling pathway. In addition, several studies found that adipose tissue sequesters vitamin D and leads to relatively low serum vitamin D levels in obese people. However, the consistency of the role of vitamin D as a mediator on the link between obesity and cancer is still low [23]. The relationship between obesity, vitamin D, and cancer risk needs to be further studied.

Numerous studies have proposed that vitamins have protective effects against breast carcinogenesis, but results have been inconsistent. Results of previous research into the roles of vitamins in breast cancer are summarized in Table 6.

In the present study, serum vitamin A and E concentrations were significantly lower in patients with breast cancer and in patients with benign breast disease than in healthy controls. Similarly, another report published in Korea reported significantly lower serum levels of carotenoid and α-tocopherol in women with breast cancer than in healthy subjects [27,28]. In addition, we found significantly lower concentrations of total cholesterol in breast cancer patients as well as benign breast disease patients than healthy controls. According to a study by Llaverias et al., plasma cholesterol levels were decreased during tumor development but not in advance to tumor initiation, indicating an increased utilization of cholesterol by tumor cells [52]. Vitamins A and E are fat-soluble vitamins that have both been reported to have significant correlations with serum cholesterol level [53,54]. Tumor demand for cholesterol may have resulted in low serum levels of total cholesterol, and therefore low levels of vitamin A and E in both benign breast disease patients and breast cancer patients in our study.

We found that the serum concentration of vitamin A was associated with a seven-fold decrease in risk of benign breast disease and a six-fold decrease in risk of breast cancer, consistent with previous findings that high levels of vitamin A were significantly associated with reduced risk of breast cancer [29,32]. Several studies have shown that a metabolite of vitamin A (all-trans retinoic acid, atRA) induces re-differentiation of transformed cells during the early stages of the neoplastic process and promotes the apoptosis of human breast cancer cells by regulating the Tet Methylcytosine Dioxygenase 2-Protein Kinase C zeta (TET2–PKCζ) pathway [55,56,57]. These results demonstrate that a low concentration of vitamin A may promote the proliferation of tumor cells in breast carcinogenesis, consistent with previous findings that breast cancer patients with a low vitamin A level tended to have advanced stage disease and a poorer prognosis than those with a high vitamin A level [24,58].

Vitamin D is the most widely investigated vitamin in terms of breast cancer [33,34,35,36,37,38,39,40,41,42,43,44,45,59]. The importance of vitamin D in breast cancer patients has been emphasized because calcitriol, the active metabolite of vitamin D, is known to have antiproliferative effects by activating apoptotic pathways and inhibiting angiogenesis [60]. Our data showed that serum vitamin D concentrations had no statistically significant differences among patients with breast cancer, patients with breast benign disease, and healthy controls. However, severe vitamin D deficiencies were more frequently found in patients with breast cancer than in healthy controls, consistent with previous studies [36,39,41,42,43]. Women with insufficient vitamin D levels have a higher risk of breast cancer and poorer overall survival than those with sufficient levels of vitamin D [33,34,36,40,59].

Low levels of vitamin B12 lead to chromosome breakage and disrupt DNA repair by influencing DNA methylation [9], although its role remains poorly understood as the published data are scarce and inconsistent [46,47,48,49,50,51]. A reduced vitamin B12 level can decrease the activity of S-adenosylmethionine (SAM) for DNA methylation and regulate gene expression, inducing breast carcinogenesis as a result [9]. We used serum methylmalonic acid and homocysteine as biomarkers of vitamin B12. Methylmalonic acid and homocysteine are likely to be more sensitive biomarkers of early vitamin B12 deficiency than direct vitamin B12 assay [61]. We observed vitamin B12 deficiency (defined as increased levels of methylmalonic acid or homocysteine) only in a couple of patients with breast cancer and patients with benign breast disease, which was statistically not significant. A study proposed by Sellers et al. revealed that women with low folate intake were at higher risk of ER-negative breast cancers because methyl deficiency induces the promotor region of the ER gene to be regionally hypermethylated, resulting in reduced protein expression [62]. Similarly, in our study, patients with triple-negative breast cancer had significantly higher methylmalonic acid levels than those with other breast cancer subtypes. However, serum concentration of methylmalonic acid is known to be influenced by multiple factors including vitamin B12 deficiency, aging, diet, or genetic mutations [63]. Further detailed studies are needed to understand the relationship between vitamin B12 and breast cancer.

In recent years, the classification of breast cancer based on histologic characteristics and gene expression has led to an improved understanding of disease pathogenesis and prognosis [42]. Triple-negative breast cancer in particular has a poor prognosis due to its aggressive behavior and lack of effective targeted therapies [18]. There have been efforts to identify risk factors for these cancers. Our findings that serum vitamin D levels were significantly lower in ER-negative and triple-negative breast cancer patients than patients with other breast cancer subtypes are in agreement with previous research [38,42,43,44,45,59]. Calcitriol affects the proliferation of breast cancer cells by regulating the expression of ERα [64,65]. This may explain the association between low vitamin D levels and aggressive breast cancers.

In our study, the concentration of vitamin E showed the strongest correlation with the concentration of total cholesterol when analyzing correlations between serum concentrations of vitamins and parameters associated with nutritional status. Vitamin E has been shown to have significant correlations with serum cholesterol, especially concentrations of lipoprotein carriers [54,66].

A strength of our study is that we measured serum levels of various vitamins in Korean breast cancer patients. Furthermore, we evaluated serum levels of these vitamins in patients with benign breast disease. We assessed the relevance of serum vitamin concentrations in accordance with molecular subtypes of breast cancer, lymph node metastasis, and tumor stages, which has only been explored previously for vitamin D. We found that concentrations of vitamins differed according to breast cancer subtypes, indicating that vitamins may have a more significant effect in breast cancers with specific characteristics. Our study suggests foundational results to support future investigations for further evidence of the roles of vitamins in breast cancer etiology. Further research evaluating relationships among serum vitamins levels, usage of dietary supplements, and treatment outcomes is needed to identify the optimal therapeutic strategies for breast cancer patients.

Our study also had several limitations. Although we evaluated several biochemical markers, information about dietary patterns (including nutrient supplements or alcohol intake) was not fully considered. Information about vitamin supplement consumption was collected via medical records, but missing data existed in some patients; thus, comprehensive interpretation may not have been provided. Vitamin supplements were taken mostly by patients with benign breast disease and patients with breast cancer. Most of the healthy controls did not take supplements (only three of them took supplements). However, each three groups of the study population showed no significant differences (*p* > 0.05) of serum vitamin concentrations between patients who took supplements and those who did not. Therefore, intake of vitamin supplements would not have affected our results. Measuring serum vitamin concentrations at a single time point may also not represent long-term vitamin condition. Future well-designed studies with large patient cohorts are needed to further explore the importance of vitamins in breast cancer.

## 5. Conclusions

In conclusion, we assessed serum concentrations of multiple vitamins or vitamin biomarkers in Korean breast cancer patients, benign breast disease patients, and healthy controls using established methodologies. Patients with breast cancer as well as patients with benign breast disease had lower concentrations of vitamins A and E, and higher frequencies of single vitamin deficiencies, including vitamin A and D, than healthy controls. Moreover, tumor subtypes known to develop more aggressively and have poorer outcomes were associated with reduced levels of vitamin D, suggesting potential subtype-specific roles and impacts of vitamins in breast cancer. Our study provides important background information regarding the potential effects of vitamins in breast cancer.

## Figures and Tables

**Table 1:** General characteristics of the study population.

		Healthy Controls (*n* = 75)	Benign Breast Disease Patients (*n* = 62)	Breast Cancer Patients (*n* = 104)	*p*-Values ^b^
Demographic characteristics					
Age (years) ^a^		49 (39–54)	43 (36–50)	49 (44–55)	0.001 ^c^
Menopausal state	Pre-menopause, N (%)	44 (58.7%)	48 (77.4%)	65 (62.5%)	0.0551
	Post-menopause, N (%)	31 (41.3%)	14 (22.6%)	39 (37.5%)	
Body mass index (kg/m^2^) ^a^		22 (20–24)	22 (20–24)	23 (21–25)	0.0059
	BMI < 23, N (%)	47 (62.7%)	43 (69.4%)	50 (48.1%)	0.0053
	23 ≤ BMI < 25, N (%)	20 (26.7%)	14 (22.6%)	29 (27.9%)	
	25 ≤ BMI, N (%)	8 (10.7%)	5 (8.1%)	25 (24.0%)	
Serum chemistry results ^a^					
Total protein (g/dL)		7.1 (6.9–7.5)	7.3 (6.8–7.5)	7.2 (6.9–7.4)	0.9314
Albumin (g/dL)		4.4 (4.3–4.6)	4.5 (4.3–4.7)	4.4 (4.3–4.6)	0.0549
AST (U/L)		19 (16–22)	19 (16–22)	17 (15–21)	0.1009
ALT (U/L)		15 (11–20)	14 (11–22)	14 (12–18)	0.8592
ALP (U/L)		55 (44–68)	54 (41–69)	56 (46–68)	0.6366
HDL (mg/dL)		63 (52–75)	64 (55–72)	63 (51–74)	0.8118
LDL (mg/dL)		116 (97–136)	100 (89–128)	108 (90–137)	0.4161
Total cholesterol (mg/dL)		190 (174–215)	167 (155–191)	178 (160–206)	0.0004 ^c^

Abbreviations: BMI, body mass index; AST, aspartate aminotransferase; ALT, alanine aminotransferase; ALP, alkaline phosphatase; HDL, high density lipoprotein; LDL, low density lipoprotein. ^a^ Results are presented as medians (interquartile ranges) or numbers (%). ^b^ *p*-values based on Kruskal–Wallis test for nonparametric data and ^c^ ANOVA for parametric data.

**Table 2:** Serum concentrations of vitamins and vitamin B12 indicators in the study population.

	Healthy Controls (*n* = 75)	Benign Breast Disease Patients (*n* = 62)	Breast Cancer Patients (*n* = 104)	*p*-Values ^b^	Healthy Controls vs. Benign Breast Disease Patients	Healthy Controls vs. Breast Cancer Patients	Benign Breast Disease Patients vs. Breast Cancer Patients
Serum Vitamin Concentrations ^a^					*p*-Values ^c^	*p*-Values ^c^	*p*-Values ^c^
Vitamin A (μmol/L)	1.76	1.35	1.45	<0.0001	<0.0001	<0.0001	0.4354
	(1.52–2.16)	(1.16–1.60)	(1.18–1.70)				
Vitamin D (ng/mL)	18.0	15.8	14.7	0.0515	0.1478	0.0546	0.9812
	(12.2–23.5)	(11.0–20.1)	(9.4–22.2)				
Vitamin E (μmol/L)	29.2	23.4	26.0	<0.0001	<0.0001	0.0006	0.1401
	(26.1–37.2)	(20.3–27.4)	(21.6–31.5)				
Methylmalonic acid (nmol/L)	111.3	112.5	96.4	0.0378	0.9923	0.0812	0.0799
	(78.5–170.4)	(80.9–165.1)	(73.5–127.3)				
Homocysteine (μmol/L)	8.0	8.2	7.8	0.8198	0.9913	0.824	0.9052
	(6.9–9.5)	(6.7–9.9)	(6.6–9.4)				

^a^ Results are presented as medians (interquartile ranges). ^b^ *p*-values are from Kruskal–Wallis test and ^c^ Tukey’s test using ranks.

**Table 3:** Crude and adjusted estimated odds ratios for breast cancer and benign breast disease (and 95% confidence intervals) associated with serum levels of vitamin status markers (multivariate logistic regression model).

		Healthy Controls (*n* = 75)	Benign Breast Disease Patients (*n* = 62)	Breast Cancer Patients (*n* = 104)
Vitamin A (μmol/L)	OR (95% CI)	1.00	0.17 (0.07–0.45)	0.26 (0.12–0.57)
	*p*-values		<0.0001	<0.0001
	OR (95% CI) ^a^	1.00	0.13 (0.04–0.36)	0.20 (0.08–0.48)
	*p*-values		0.0001	0.0004
Vitamin D (ng/mL)	OR (95% CI)	1.00	0.99 (0.95–1.03)	0.99 (0.96–1.03)
	*p*-values		0.1478	0.0546
	OR (95% CI) ^a^	1.00	0.98 (0.94–1.02)	0.99 (0.95–1.02)
	*p*-values		0.3441	0.4358
Vitamin E (μmol/L)	OR (95% CI)	1.00	0.92 (0.87–0.98)	0.96 (0.92–1.01)
	*p*-values		<0.0001	0.0006
	OR (95% CI) ^a^	1.00	0.97 (0.90–1.05)	0.96 (0.91–1.02)
	*p*-values		0.4428	0.1867
Methylmalonic acid (nmol/L)	OR (95% CI)	1.00	1.00 (1.00–1.01)	1.00 (0.99–1.00)
	*p*-values		0.9923	0.0812
	OR (95% CI) ^a^	1.00	1.01 (1.00–1.01)	1.00 (0.99–1.01)
	*p*-values		0.1169	0.6642
Homocysteine (μmol/L)	OR (95% CI)	1.00	1.01 (0.85–1.20)	1.01 (0.87–1.16)
	*p*-values		0.9913	0.8240
	OR (95% CI) ^a^	1.00	1.03 (0.86–1.23)	0.99 (0.84–1.17)
	*p*-values		0.7615	0.8916

Abbreviations: OR, odds ratio; 95% CI, 95% confidence interval. ^a^ Adjusted for age, menopausal state, BMI, total protein, albumin, AST, ALT, ALP, and total cholesterol.

**Table 4:** Subgroup analyses of vitamin levels in 104 breast cancer patients ^a^.

		Vitamin A (μmol/L)	Vitamin D (ng/mL)	Vitamin E (μmol/L)	Methylmalonic Acid (μmol/L)	Homocysteine (μmol/L)
ER	Negative (*n* = 12)	1.34	8.5	27.3	104.8	8.1
		(1.16–1.67)	(7.1–15.8)	(22.3–29.0)	(85.8–163.4)	(6.5–9.6)
	Positive (*n* = 92)	1.45	15.0	25.8	95.4	7.8
		(1.19–1.72)	(10.1–22.7)	(21.6–32.0)	(73.0–124.5)	(6.7–9.4)
	*p*-values ^b^	0.6145	0.0233	0.9959	0.2164	0.9756
PR	Negative (*n* = 19)	1.48	13.5	27.1	96.8	8.6
		(1.13–1.86)	(8.0–22.9)	(21.9–29.1)	(84.0–173.4)	(7.2–10.2)
	Positive (*n* = 85)	1.44	14.7	25.6	94.0	7.7
		(1.20–1.63)	(9.8–22.0)	(21.6–32.1)	(73.1–122.6)	(6.6–9.3)
	*p*-values ^b^	0.7144	0.6346	0.9062	0.2778	0.1288
HER2	Negative (*n* = 76)	1.45	14.3	26.0	94.7	7.7
		(1.19–1.70)	(9.1–21.9)	(21.7–31.6)	(73.0–125.7)	(6.5–9.2)
	Positive/Equivocal (*n* = 28)	1.46	16.3	26.2	96.4	8.4
		(1.14–1.73)	(10.2–24.6)	(20.8–31.2)	(75.3–135.4)	(7.2–10.2)
	*p*-values ^c^	0.9357	0.5001	0.6788	0.8032	0.0946
TN	No (*n* = 95)	1.45	15.0	26.0	94.3	7.9
		(1.17–1.69)	(9.8–22.5)	(21.6–31.7)	(73.1–124.4)	(6.6–9.4)
	Yes (*n* = 6)	1.30	8.5	25.7	152.2	7.5
		(1.18–1.71)	(7.2–11.3)	(21.9–29.1)	(113.4–177.7)	(6.5–8.3)
	*p*-values ^b^	0.6011	0.0266	0.775	0.0384	0.4641
LN metastasis	No (*n* = 88)	1.45	15.0	25.8	94.3	7.8
		(1.19–1.68)	(9.3–22.6)	(21.7–31.5)	(73.3–126.0)	(6.7–9.3)
	Yes (*n* = 16)	1.38	13.4	26.6	108.3	8.8
		(1.14–1.93)	(9.7–20.3)	(20.5–31.7)	(80.7–134.5)	(6.5–10.3)
	*p*-values ^b^	0.8429	0.6654	0.9139	0.3605	0.502
Stage	Stage 0~1/LCIS (*n* = 77)	1.44	14.7	25.6	94.6	7.8
		(1.18–1.67)	(9.0–22.3)	(21.6–31.3)	(73.4–127.6)	(6.6–9.2)
	Stage 2 (*n* = 19)	1.48	16.0	25.1	103.3	8.7
		(1.28–1.86)	(9.3–22.8)	(21.6–30.2)	(82.5–131.0)	(6.8–10.2)
	Stage 3 (*n* = 8)	1.19	14.0	31.7	94.3	7.9
		(1.04–1.89)	(11.3–18.5)	(22.9–35.8)	(75.0–110.5)	(6.5–9.6)
	*p*-values ^c^	0.5472	0.8976	0.447	0.6803	0.5515

Abbreviations: ER, estrogen receptor; PR, progesterone receptor; HER2, human epidermal growth factor receptor 2; TN, triple-negative; LN, lymph node; LCIS, lobular carcinoma in situ. ^a^ Results are presented as medians (interquartile ranges). ^b^ *p*-values estimated through Wilcoxon rank sum test and ^c^ Kruskal–Wallis test.

**Table 5:** Correlations among vitamin status, basal characteristics, and biochemical parameters of the study population ^a^.

	Age	BMI	TP	Albumin	AST	ALT	ALP	HDL	LDL	TC
Vitamin A	0.298 ^c^	0.093	−0.110	−0.023	0.257 ^c^	0.261 ^c^	0.182 ^c^	−0.026	0.096	0.183 ^c^
Vitamin D	0.156 ^b^	−0.08	−0.032	0.059	0.196 ^c^	0.177 ^c^	0.026	0.096	−0.015	0.015
Vitamin E	0.292 ^c^	0.101	0.062	−0.027	0.153 ^b^	0.186 ^c^	0.067	0.119	0.421 ^c^	0.586 ^c^
Methylmalonic acid	0.164 ^b^	−0.029	−0.122	−0.067	0.042	−0.027	0.119	0.026	0.027	0.064
Homocysteine	0.206 ^c^	0.137 ^b^	0.009	−0.105	0.052	0.005	0.207 ^c^	−0.159 ^b^	0.089	0.093

Abbreviations: BMI, body mass index; TP, total protein; AST, aspartate aminotransferase; ALT, alanine aminotransferase; ALP, alkaline phosphatase; HDL, high density lipoprotein; LDL, low density lipoprotein; TC, total cholesterol. ^a^ Results are described as Spearman’s correlation coefficients. ^b^ *p*-values < 0.05. ^c^ *p*-values < 0.01.

**Table 6:** A summary of previous studies of vitamin status in patients with breast cancer.

Studied Vitamins	Region	Numbers (Cases/Controls)	Analytes	Results	References
Vitamin A	Italy	208	Retinol (μmol/L)	BC patients (≥55 year-old) with low retinol levels had a poorer prognosis (hazard ratio = 3.58, 95% CI = 1.50–8.57).	Formelli, 2009 [24]
Vitamin E	India	75/75/50 ^a^	α-tocopherol (ug/mL)	BC and BBD patients had significantly lower vitamin E levels (*p* < 0.001), and decreased vitamin E was directly related to higher stage BC.	Chitkara, 1996 [25]
Vitamin A and E	USA	105/203	Retinol (μmol/L), α-tocopherol (μmol/L)	No evidence for protective effects of α-tocopherol or retinol in BC.	Dorgan, 1998 [26]
Vitamin A and E	Sweden	201/290	Retinol (μmol/L), α-tocopherol (μmol/L)	No significant associations between plasma levels of α-tocopherol or retinol and BC risk.	Hultén, 2001 [27]
Vitamin A and E	Korea	160/229	Retinol (μg/dL), α-tocopherol (μg/mL)	Significantly lower α-tocopherol and retinol levels in BC patients than in controls (*p* < 0.001). Significantly decreased BC risks with increasing α-tocopherol and retinol levels (α-tocopherol, OR = 0.13, 95% CI = 0.03–0.66; Retinol, OR = 0.08, 95% CI = 0.01–0.45)	Kim, 2001 [28]
Vitamin A and E	Australia	153/151	Retinol (μmol/L), α-tocopherol (μmol/L)	Significant reduction of BC risk with increasing retinol levels (OR = 0.53, 95% CI 0.28–1.01, *p* = 0.04), but no significant association with BC risk and α-tocopherol levels (OR = 1.27, 95% CI = 0.69–2.35, N.S.)	Ching, 2002 [29]
Vitamin A and E	USA	969/969	Retinol (μmol/L), α-tocopherol (μmol/L)	Retinol (*p* = 0.03) and α-tocopherol (*p* = 0.01) levels were associated with a significantly decreased risk of BC with LN metastasis.	Tamimi, 2005 [30]
Vitamin A and E	France	366/720	Retinol (μmol/L), α-tocopherol (μmol/L)	No significant associations between BC risk and serum carotenoids (OR = 0.74, 95% CI = 0.47–1.16, N.S.), α-tocopherols (OR = 0.70, 95% CI = 0.44–1.13, N.S.), or retinol (OR = 0.85, 95% CI = 0.53–1.35, N.S.) in postmenopausal women.	Maillard, 2010 [31]
Vitamin A and E	Korea	376/304	Retinol (μg/dL), α-tocopherol (μg/dL)	Higher retinol level was associated with lower BC risk (OR = 0.13, 95% CI = 0.07–0.26), but this was not true for α-tocopherol level.	Kim, 2010 [32]
Vitamin D	USA	701/724	25(OH)D (ng/mL), 1,25(OH)_2_D (ng/mL)	High levels of vitamin D were associated with lower BC risk, but this was not statistically significant (25(OH)D, RR = 0.73, 95% CI = 0.49–1.07, N.S.; 1,25(OH)_2_D, RR = 0.76, 95% CI = 0.52–1.11, N.S.).	Bertone-Johnson, 2005 [33]
Vitamin D	Germany	1394/1365	25(OH)D (nM)	Significant inverse association between vitamin D levels and post-menopausal BC risk (OR = 0.31, 95% CI = 0.24–0.42, *p* < 0.0001).	Abbas, 2008 [34]
Vitamin D	USA	1005/1005	25(OH)D (ng/mL), 1,25(OH)_2_D (pg/mL)	No inverse association between vitamin D levels and BC risk (25(OH)D, RR = 1.04, 95% CI = 0.75–1.45, N.S.; 1,25(OH)_2_D, RR = 1.23, 95% CI = 0.91–1.68, N.S.).	Freedman, 2008 [35]
Vitamin D	USA	1026/1075	25(OH)D (ng/mL)	Mean vitamin D levels were significantly lower in BC patients than in controls (*p* < 0.0001). There was an inverse association between vitamin D and BC risk in a concentration-dependent manner (*p* = 0.002).	Crew, 2009 [36]
Vitamin D	Sweden	764/764	25(OH)D_2_ (nmol/L), 25(OH)D_3_ (nmol/L)	Weak inverse association between 25(OH)D_3_ levels and BC risk, but this was not statistically significant. There was a weaker association between total 25(OH)D (25(OH)D_2_ + D_3_) and BC.	Almquist, 2010 [37]
Vitamin D	USA	579/574	25(OH)D (ng/mL)	Significantly lower vitamin D levels in BC patients (*p* < 0.001), and lower vitamin D levels in high grade BC, including ER(-) tumors (*p* ≤ 0.03) and TNBC (*p* = 0.002).	Yao, 2011 [38]
Vitamin D	Korea	310 ^b^	25(OH)D (ng/mL)	Vitamin D deficient individuals (<20 ng/mL) had increased risk of recurrence compared with those with sufficient vitamin D levels (30–150 ng/mL) (*p* = 0.002). Inverse association between vitamin D levels and prognosis of BC in luminal A (*p* = 0.012) and luminal B subtypes (*p* = 0.023), but no association with prognosis of BC in HER2(+) or TN subtypes.	Kim, 2011 [39]
Vitamin D	Germany	1295 ^b^	25(OH)D (nmol/L)	BC patients with lower vitamin D levels had a higher risk of death (hazard ratio = 1.08, 95% CI = 1.00–1.17) and significantly higher risk of distant recurrence (hazard ratio = 1.14, 95% CI = 1.05–1.24).	Vrieling, 2011 [40]
Vitamin D	Pakistan	90/90	25(OH)_2_D (ng/mL)	Significantly lower vitamin D levels in BC patients than in controls (*p* < 0.001). However, no significant association between tumor characteristics and vitamin D levels among BC patients.	Imtiza, 2012 [41]
Vitamin D	USA	194/194	25(OH)D (ng/mL)	Significantly lower vitamin D levels in BC patients than in controls (*p* = 0.02). Patients with suboptimal vitamin D levels (<32 ng/mL) had significantly higher risk of having ER(-) (OR = 2.59, 95% CI = 1.08–6.23) and TN (OR = 3.15, 95% CI = 1.05–9.49) BC than those with optimal vitamin D levels (≥32 ng/mL). BC patients with a basal-like subtype had lower vitamin D levels than BC patients with a luminal subtype (*p* = 0.04).	Peppone, 2012 [42]
Vitamin D	Korea	3634/17,133	25(OH)D (ng/mL)	Significantly higher BC risk in women with vitamin D deficiency than those with sufficient vitamin D (OR = 1.27, 95% CI = 1.15–1.39). Significant inverse association between vitamin D levels and HR(-) BC, particularly TNBC (OR = 1.45, 95% CI 1.15–1.82).	Park, 2015 [43]
Vitamin D	Sweden	764/764	25(OH)D_3_ (nmol/L)	Women with vitamin D levels of ≥77 but ≤97 nmol/L had a significantly lower risk of an ER(-) tumor (OR = 0.46, 95% CI = 0.23–0.94), PR(-) (OR = 0.66, 95% CI = 0.46–0.96) and higher Ki67 tumor expression (OR = 0.57, 95% CI = 0.36–0.90) than those with vitamin D levels of ≤76 nmol/L.	Shirazi, 2016 [44]
Vitamin D	Brazil	192 ^b^	25(OH)D (ng/mL)	Patients insufficient (20–29 ng/mL) or deficient (<20 ng/mL) for vitamin D had higher proportions of high-grade BC, advanced BC, metastatic disease, number of positive LNs, and high Ki-67 expression in their tumors (*p* < 0.05).	de Sousa Almeida-Filho, 2017 [45]
Vitamin B12	USA	195/195	Folate (ng/mL), Vitamin B12 (pg/mL) Homocysteine (nmol/mL)	Significantly lower vitamin B12 levels in BC in postmenopausal women (*p* = 0.03). Women with lower vitamin B12 levels showed increased BC risk (OR = 4.00, 95% CI = 1.05–15.20). No association between folate and homocysteine and BC risk.	Wu, 1999 [46]
Vitamin B12	USA	712/712	Folate (ng/mL), Vitamin B12 (pg/mL), Homocysteine (nmol/mL)	Significantly lower folate concentrations in BC than in controls (*p* = 0.009). Inverse association between vitamin B12 levels and risk of BC only among premenopausal women (RR = 0.36, 95% CI = 0.15–0.86). No association between homocysteine and BC risk.	Zhang, 2003 [47]
Vitamin B12	USA	848/848	Folate (ng/mL), Vitamin B12 (pg/mL)	No significant association between folate and vitamin B12 levels and overall risk of BC. Higher folate levels were associated with an increased risk of premenopausal BC (*p* = 0.04).	Lin, 2008 [48]
Vitamin B12	USA	812/812	Cysteine (nmol/mL), Homocysteine (nmol/mL)	Positive association between cysteine levels and BC risk (RR = 1.65, 95% CI = 1.04–2.61, *p* = 0.04). Patients who had low folate levels tend to have positive correlations between concentrations of homocysteine and cysteine and risk of BC development (*p* = 0.04 and 0.002). No association between homocysteine and overall BC risk.	Lin, 2010 [49]
Vitamin B12	Canada	164 ^b^	Folate (ng/mL), Vitamin B12 (pmol/L)	Significant association between high plasma folate levels (>24.4 ng/mL) and increased BC risk (HR = 3.20, 95% CI = 1.03–9.92, *p* = 0.04) than low folate (≤24.4 ng/mL). No significant association between vitamin B12 concentration and BC risk.	Kim, 2016 [50]
Vitamin B12	USA	610/1207	Folate (ng/mL), Vitamin B12 (pg/mL), Homocysteine (nmol/mL)	Plasma vitamin B12 was positively associated with higher risk of overall BC (95% CI = 1.17–2.29, *p* = 0.02), and plasma folate was positively associated with risk of invasive BC.	Houghton, 2019 [51]

Abbreviations: BC, breast cancer; CI, confidence interval; BBD, benign breast disease; OR, odds ratio; N.S., not significant; LN, lymph node; RR, relative risk; ER, estrogen receptor; (−), negative; TNBC, triple-negative breast cancer; HER2, human epidermal growth factor receptor 2; (+), positive; TN, triple-negative; HR, hormone receptor; PR, progesterone receptor. ^a^ Breast cancer patients/benign breast disease patients/healthy controls. ^b^ Only patients with breast cancer were included in the study.

## Supplementary Materials

The following are available online at https://www.mdpi.com/2072-6643/12/9/2831/s1. Figure S1: An example of multiple reaction monitoring (MRM) chromatograms of analytes (left) and its internal standards (right) in liquid chromatography with tandem mass spectrometry (LC-MS/MS) analysis. (**a**) 25-hydroxyvitamin D2 and (**b**) 25-hydroxyvitamin D3.
