# Supplementary material for: Serum Vitamin Levels and Their Relationships with Other Biomarkers in Korean Breast Cancer Patients
Source: Nutrients. 2020 Sep 16;12(9):2831. doi: 10.3390/nu12092831 (PMC7550996; doi:10.3390/nu12092831)
Supplement: Supplementary file 1 [file nutrients-12-02831-s001.pdf]

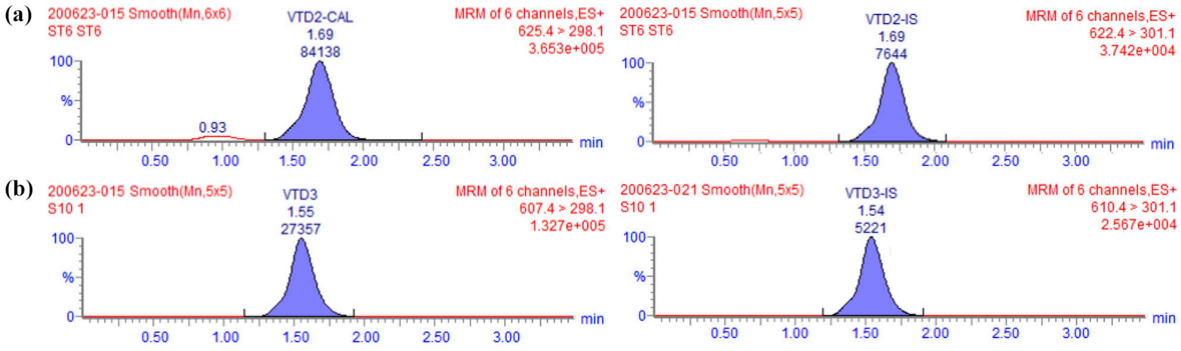

Figure S1. An example of multiple reaction monitoring (MRM) chromatograms of analytes (left) and its internal standards (right) in liquid chromatography with tandem mass spectrometry (LC-MS/MS) analysis. (**a**) 25-hydroxyvitamin D2 and (**b**) 25-hydroxyvitamin D3.
